# Interaction of Background Noise and Auditory Hallucinations on Phonemic Mismatch Negativity (MMN) and P3a Processing in Schizophrenia

**DOI:** 10.3389/fpsyt.2020.540738

**Published:** 2020-09-15

**Authors:** Ashley M. Francis, Verner J. Knott, Alain Labelle, Derek J. Fisher

**Affiliations:** ^1^ Department of Psychology, Saint Mary’s University, Halifax, NS, Canada; ^2^ Royal Ottawa Mental Health Centre, Ottawa, ON, Canada; ^3^ Department of Psychology, Carleton University, Ottawa, ON, Canada; ^4^ Department of Psychology, Mount Saint Vincent University, Halifax, NS, Canada

**Keywords:** schizophrenia, hallucinations, mismatch negativity, electroencephalography, ERP, event-related potentials, P3a, P300

## Abstract

Auditory hallucinations (AHs) are among the cardinal symptoms of schizophrenia (SZ). During the presence of AHs aberrant activity of auditory cortices have been observed, including hyperactivation during AHs alone and hypoactivation when AHs are accompanied by a concurrent external auditory competitor. Mismatch negativity (MMN) and P3a are common ERPs of interest within the study of SZ as they are robustly reduced in the chronic phase of the illness. The present study aimed to explore whether background noise altered the auditory MMN and P3a in those with SZ and treatment-resistant AHs.

**Methods:**

MMN and P3a were assessed in 12 hallucinating patients (HPs), 11 non-hallucinating patients (NPs) and 9 healthy controls (HCs) within an auditory oddball paradigm. Standard (P = 0.85) and deviant (P = 0.15) stimuli were presented during three noise conditions: silence (SL), traffic noise (TN), and wide-band white noise (WN).

**Results:**

HPs showed significantly greater deficits in MMN amplitude relative to NPs in all background noise conditions, though predominantly at central electrodes. Conversely, both NPs and HPs exhibited significant deficits in P3a amplitude relative to HCs under the SL condition only.

**Significance:**

These findings suggest that the presence of AHs may specifically impair the MMN, while the P3a appears to be more generally impaired in SZ. That MMN amplitudes are specifically reduced for HPs during background noise conditions suggests HPs may have a harder time detecting changes in phonemic sounds during situations with external traffic or “real-world” noise compared to NPs.

## Introduction

### Schizophrenia

Schizophrenia (SZ) is a debilitating neurological disease (1) that affects approximately 1% of the world’s population (2), with the global prevalence of SZ rising by over 5% percent in 26 years (3). SZ is a multifaceted disease that affects one’s behavior, cognition, and intellectual functioning. Among the cardinal symptoms of SZ are auditory hallucinations (AH), being reported in 50%–80% of diagnosed cases (4–10).

AHs are described as an aural sensory experience occurring during an awake state in the absence of external stimulation, over which the individual feels they have no control and which have a sense of realism (10, 11). AHs are believed to be a consequence of disruptions in the metacognitive processes that categorizes self-generated and external information sources, in addition to further deficits in information processing (12–15). The exact cause and mechanism of AHs, however, still remain unclear (16). It has been suggested that AHs may compete against external auditory stimuli for attentional resources (17–19); this is supported by the findings that AHs and auditory cortex activity are diminished in cases where external speech competitors are presented (20–22). While AHs have been explored using many different methodologies, electroencephalography (EEG) has proven effective at capturing concurrent rapid changes in cortical activity (23).

### EEG

EEG is a non-invasive procedure that measures the summation of the neural activity of large groups of neurons firing; Event-related potentials (ERPs) are derived from EEG by assessing neuroelectric activity elicited simultaneously in response to stimuli (e.g., light or tones). ERPs are an exceptionally sensitive method to index cognition due to their temporal sensitivity and can be used to help supplement and comprehend behavioral observations.

### MMN

Within SZ, one of the most commonly studied ERPs is the auditory mismatch negativity [MMN; (24–26)]. MMN is well suited in that it does not require a behavioral response by the participant (27). MMN is elicited by any detectable change in an auditory stimulus, occurring regardless of conscious awareness to the change (28, 29). MMN is a fronto-central negatively peaking waveform with a typical latency of 90–250 ms (29–31). It was previously suggested that this waveform is produced when there is a discriminatory difference between the auditory stimuli presented and the memory trace of previous stimuli (29). The more recent prediction error models of the MMN suggest this waveform is elicited when the incoming stimulus violates a prediction based on a sensory model of the current auditory environment (32, 33).

MMN amplitude has been shown to be robustly reduced in individuals with SZ when compared to healthy controls (HCs) (34, 35). It appears MMN deficits are particularly robust in SZ among other psychotic disorders. Previous work has reported MMN amplitude reductions were most prominent in patients with SZ when compared to individuals with bipolar disorder (36, 37). Previous studies measuring MMN alterations in major psychiatric disorders (e.g., depression, obsessive-compulsive disorder) have found similar findings, suggesting the decreased amplitude in MMN may be specific to the SZ population (38–41).

MMN has also been found to index brain deterioration in the SZ clinical populations (36, 42–44). MMN amplitude reduction has been conceptualized to be associated with gray matter loss in the supra-temporal cortex, as well as widespread loss across the cortex (37, 45, 46). In addition, greater reductions of MMN amplitudes have been observed in later stages of illness (47–49). The reduction of MMN amplitudes can in part be attributed to the deficient functioning of *N-*methyl-D-aspartate (NMDA) glutamate receptor (50–52).

Given the heterogeneity of SZ, there has been interest in how specific symptoms, such as AHs, contribute to brain-based changes in this illness (23). Previous research has found a link between severity of AH trait (i.e., predisposition to experience AHs) and MMN amplitude deficits (23, 53, 54), suggesting that an increase in AH trait is associated overall decrease in MMN amplitude (53–55). The link between AH trait, the MMN and neuroanatomy has been previously reported by Salisbury et al. (37, 56), who reported that gray matter loss near Heschl’s gyrus is associated with both an overall increase in AHs and a decrease in MMN amplitude. While increased hallucinatory trait has been linked to MMN deficits, the link between AH state and MMN (i.e., MMN amplitudes being reduced during a concurrent AH experience) is significantly more tenuous. This suggests that reductions in MMN amplitude are associated with brain-based changes associated with the production of hallucinations, rather than AHs usurping auditory processing resources within a limited capacity system.

### P3a

The P300a, or P3a, is an ERP that often follows the MMN (57) and is often observed between 200 and 650 ms. P3a is a positive waveform with a stereotypical fronto-central scalp distribution (58–60). The auditory P3a is conceptualized as the directing of one’s attention to a deviant or novel sound (59, 61). Additionally, P3a can be elicited when there is a particular significance to the auditory stimulus either socially or emotionally, both of which are related to pre-existing information in long term memory (57).

Commonly the P3a is elicited in experimental research through an active three-stimulus auditory oddball paradigm, in which the participant is presented with a string of standard stimulus with non-target distractors, and target tones for which they are told to identify (61–63). P3a can, however, be elicited through the means of a passive 2- or 3-stimulus-auditory oddball-paradigm (63–66).

Reductions in P3a amplitude have been shown in individuals with chronic SZ (64, 67), after their first episode of psychosis (68–70) as well as in individuals classified as being high risk for developing SZ (71–73). This evidence suggests that P3a could serve as an important marker of SZ, including for those at risk of developing SZ. P3a is elicited in a similar manner to MMN whereby a combination of standard and deviant auditory stimuli is presented passively. P3a is elicited when attention is directed at a deviating tone. While MMN and P3a are associated, evidence shows that they are not dependant on each other and can occur in absence of one another (57, 74, 75).

While pure tone stimuli are typically used to elicit the MMN and P3a, both can be elicited by any auditory deviant, including spectrally-complex phonetic stimuli (76–78). Numerous studies have examined MMNs elicited by speech sounds to probe language-based processes in healthy and clinical populations (52, 74, 74, 76, 79, 80), with many employing phonemes (i.e., the simplest unit of speech with linguistic meaning) as standard and deviant stimuli. The earliest study to use a phonemic paradigm to investigate the MMN in SZ reported significant deficits in MMN amplitudes elicited by a cross-phoneme change in SZ patients [vs. HCs; (81)]. In a follow-up study examining phonemic MMN in SZ patients with AHs, SZ patients with no hallucinations, and HCs (54), SZ patients were found to have significant deficits in MMN amplitudes when compared to HCs; however, no significant differences were found between the two patients subgroups (54).

It is speculated that P3a is altered by neurobiological instabilities such as those shown in SZ (26, 66) while other studies have shown a reduction in P3a amplitudes being linked to increased disease duration and an increase in AHs (82, 83). Of the studies that have measured P3a with a SZ sample, reductions in P3a amplitude *via* the traditional oddball paradigm (73, 84–86) have been previously reported as well as P3a amplitude reductions when using a phonetic paradigm (77). A study measuring P3a amplitude and latency with the traditional oddball phoneme paradigm in HCs showed that P3a amplitude and latency were correlated with recall of verbal information and working memory performance (66), similar to what was found for MMN. To date, little research has been done to measure P3a amplitudes in SZ with and without AHs. The literature that does exist shows those with AH had decreased P3a amplitudes compared to those without AHs and HCs (77).

### Objectives and Hypotheses

The current study aimed to expand our understanding of the neurological workings of those with SZ, and elaborate on how AHs affect auditory processing by studying a group of individuals who report experiencing AHs and a group who do not. In order to better understand how auditory functions may change under different auditory conditions, MMN and P3a were examined under different background noise conditions [white noise (WN), “real-life” traffic noise (TN), and silence (SL)]. Compared to HCs it is expected that both patient groups will have significant reductions in both the MMN and P3a amplitudes; furthermore, it is expected that individuals currently experiencing AH will show the greatest deficits in MMN and P3a generation. Finally, we hypothesize that MMN and P3a will be reduced under conditions of auditory competition (TN and WN) compared to SL in all groups.

## Methods

### Study Participants

#### Experimental Participants

Experimental participants were comprised of twenty-four individuals between the ages of 18–65 presenting with a primary diagnosis of SZ. Participants were recruited through the Outpatient Schizophrenia Clinic of the Royal Ottawa Mental Health Centre in Ottawa Ontario. Data from these participants in response to different auditory paradigms have previously been reported (54, 77, 87); none of the neurophysiological data reported herein has previously been published.

Participants were assessed by their primary care physician with respect to inclusion/exclusion criteria for the study and had to be deemed as stable for four weeks prior to testing. Clinical history and ratings for the Positive and Negative Syndrome Scale (PANSS) were used to classify participants into the hallucinating (HP) or non-hallucinating (NP) condition. HPs (n = 12) were patients who reported a certain and consistent history of AHs over the course of their illness history. HPs exhibited a score of ≥3, which equates to a mild or greater hallucinatory experience on the hallucination item of the PANSS positive symptom scale. NPs (n = 12) were patients displaying a score of 1 on the same hallucination item of the PANSS and displaying no previous consistent AH history.

Confirmation of AHs was completed upon arrival to the laboratory through the Psychotic Symptom Rating Scale [PSYRATS; (88)]. The PSYRATS allowed us to quantify the frequency, duration, and loudness as well as other aspects of the AHs. HP and NPs were equivalent on age, gender, PANSS score [Positive Scale, Negative Scale and General Psychopathology Scale; (89)] and medication dosage (converted to chlorpromazine equivalents; CPZ). All participants had to present with normal hearing according to the audiometric assessment conducted on the study day requiring thresholds of 25 dB (SPL) or less to pure tones of 500, 1,000, and 2,000 Hz. A summary of participant demographic data can be found in **Table 1**.

**Table 1:** Summary of participant demographic data (mean ± SD).

	HP	NP	HC
Age	44.25 (10.90)	45.09 (10.70)	40.44 (12.96)
Sex male (female)	8(4)	9(2)	4(5)
Rx in CPZ equivalent (mg)	500.00 (231.60)	327.27 (161.42)	–
PANSS positive*	16.45 (3.40)	11.18 (4.31)	–
PANSS negative	14.45 (4.21)	13.27 (5.24)	–
PANSS general*	27.73 (6.38)	21.18 (4.51)	–
PANSS hallucination item*	3.64 (0.64)	1.00 (0)	–
PSYRATS	28.67 (4.16)	–	–

*significant difference between groups (p <.05).

##### Exclusion Criteria

Participants were excluded if they met any of the following: Co-morbid DSM- IV TR Axis I disorder; total PANSS score >65; present account of drug abuse/dependence; history of head injury; diagnosis of epilepsy or any other form of neurological disorder; treatment using electroconvulsive therapy (ECT) within the previous year; extrapyramidal symptoms (EPS); significant cardiac illness; or abnormal audiometric assessment.

#### Control Participants

HCs were 12 volunteers who displayed normal hearing. As was the case with the clinical participants, data from these unaffected controls recorded in response to different auditory paradigms have previously been reported (54, 77, 87). Participants were required to self-report psychiatric, medical, neurological, and alcohol/drug use/abuse histories as well as no current regular medication use (with the exception of oral contraceptives). Controls were matched to the experimental sample on measures of age, gender, and NART scores.

### ERP Recording

ERPs of interest were extracted from the EEG activity, which was recorded for each participant using an electrode cap with Ag^+^/Ag^+^-Cl^−^ ring electrodes at 32 scalp sites according to the 10–20 system of electrode placement, including three midline sites [frontal (F_z_), central (C_z_), parietal (P_z_)], three left hemisphere [frontal (F_3_), central (C_3_), temporal (T_7_)] and three right hemisphere [frontal (F_4_), central (C_4_), temporal (T_8_)]. Electrodes were also placed on right and left mastoid, as well as nose and mid-forehead to serve as ground and reference channels respectively. Electro-oculogram activity was recorded from supra-/sub-orbital and external canthi sites *via* bipolar channels. All electrode impedances were below 5 kΩ; all electrical activity was recorded using BrainVision Recorder software with an amplifier bandpass setting of 0.1-30 Hz and digitized at 500 Hz. Data was then stored on a hard-drive for offline analysis using the BrainVision Analyzer software.

### Neurophysiological Battery

MMN and P3a were assessed during three background conditions: SL, wide-band WN, and TN. The consonant-vowel (CV) syllables used to elicit MMN and P3a activity was identical to that of (90). The standard “ba’” (510 stimuli; P = 0.85) and deviant “da” (90 stimuli; P = 0.15) phonemes were presented in one block of 600 stimuli for each condition; presentation orders of background noise conditions (TN, SL, and wideband WN) were randomized and counter-balanced using a Latin square design. Stimuli were presented with an interstimulus interval (ISI) of 550–600 ms in a pseudo-randomized order ensuring that there was a minimum of three standard tones between each deviant.

The CV stimuli were created by having a male speak into a studio microphone and digitizing the audio stimuli using the Audacity program (22-kHz sampling rate, 11-kHz anti-aliasing filter, 12 dB/octave); edited to 150-ms duration. Spectral analysis of the CV stimuli showed them to display most of their power in the 100–2,000 Hz range, with very little power above 4,000 Hz. This was confirmed by Fast Fourier Transform (FFT) analysis, which showed most of the auditory energy to exist in the 100–2,000 Hz range in both CVs.

The WN and TN backgrounds were created within the hospital Audio-Visual department by digitizing from a pre-recorded sound effects audio tape and transferring ~12-min segments of each noise type to (CD) disc. Background noise was presented through external speakers and computer-presented CV stimuli (delivered using a digital-to-audio sampling rate of 16 kHz) were delivered binaurally through headphones, with the peak intensity of the CV signals being 10-dB sound pressure level (SPL), higher than the background noise intensity of 60 dB (SPL). Therefore, the signal-to-noise ratio (SNR) was +10 dB in the combined conditions. While the stimuli were presented, participants were asked to watch a silent, neutral emotive video and ignore the auditory phoneme stimuli being presented; each block lasted approximately 6 min.

Following each block, participants were asked to rate the hallucinations experienced during the recordings on the following three dimensions; 1) duration (0 = no AHs; 7 = continuous AHs); 2) loudness (0 = not audible; 7 = extremely loud); and 3) clarity (0 = unintelligible; 7 = very clear). See **Table 2** for mean hallucination scores for each noise condition.

**Table 2:** Mean (± SE) state hallucination ratings separated by noise conditions.

State Hallucination rating	Noise condition		
	Silence	Traffic Noise	White Noise
Duration	2.08 (0.57)	2.83 (0.61)	2.50 (0.57)
Loudness	1.50 (0.29)	1.75 (0.30)	1.91 (0.45)
Clarity	1.75 (0.54)	1.83 (0.40)	2.08 (0.54)

### EEG Data Processing

Data from all 32 scalp sites was re-referenced from a common reference to the nose channel before being segmented into standard and deviant tones for each of the three noise conditions (SL, TN, and WN). Digital filters were then applied using a band pass of 0.15–20 Hz (91). Electrical epochs (500-ms period, commencing 100-ms pre-stimulus) were corrected for eye movement (residual movement and blinks) using the Gratton & Coles algorithm (92), and baseline corrected using the pre-stimulus period (i.e., −100–0 ms); following these corrections, epochs exceeding ± 100 µV were excluded from further analysis. Following this, the data was put through an artifact rejection process whereby any epochs with data exceeding a 20µV/ms voltage step or exceeding ±75 µV within the epoch were excluded and the data was then averaged. Difference waves were generated by subtracting the waveforms of the standard stimulus away from waveforms generated by the deviant stimulus for each of the three conditions.

#### MMN Processing

MMN waveforms were individually assessed in difference waves within a custom selection window from 100 to 270 ms; this window was chosen by observing the grand average of the data. MMN peaks were picked as the most negative point within the window and the output was the average within five voltage points (10 ms) to the left and right of the peaks amplitude. MMN amplitude and latency were measured at scalp site F_z_ which is the site of maximum amplitude.

#### P3a Processing

P3a was analyzed through similar means and a peak detection window of 200-450 ms was identified for the difference waveforms this window was chosen by observing the grand average of the data. P3a was then picked as the largest positive peak within the given window of time. P3a amplitudes were quantified as the average within five voltage points (10 ms) to the right and left of the positive peak. P3a latency and amplitude were measured at C_z_, as this was the site of maximum amplitude.

### Study Procedure

Participants completed informed consent before attending the laboratory session. Following the completion of informed consent, participants were asked to complete demographic information. In addition, participants were measured on general medical/health, handedness [Edinburg Handedness Inventory; (93)], the National Adult Reading Test [NART; (94)] which provides an approximation of premorbid Full-Scale I.Q. Upon arrival to the laboratory, EEG electrodes were applied and participants completed the experiment.

Testing took place between 11 am and 2 pm and participants were asked to refrain from drug use (tobacco, alcohol, and cannabis), and medication (with the exception of anti-psychotics and adjunct drugs) beginning at midnight the night before their testing session.

Study procedures were conducted following clearance by both the Royal Ottawa Mental Health Center and Carleton University (CU) Research Ethics Boards.

### Statistical Analysis

Some participants had to be excluded following data analysis due to uninterpretable data. More specifically, three HCs and one non-hallucinating participant were lost, leaving final groups of n = 12 for HPs, n = 11 for NPs, and n = 9 for HCs.

Analysis was carried out by using the Statistical Package for the Social Sciences (SPSS; SPSS Inc., Chicago, IL). MMN and P3a amplitudes were assessed with mixed analysis of variance (ANOVA), with one between group measure (3 levels: HC, HP, and NP) and three-within group factors 1. Noise (3 levels: SL, TN, WN), 2. Site (3 levels: left, midline, right), and 3. Region (2 levels: Frontal and Central). *A priori* planned comparisons were conducted to quantify MMN and P3a amplitude differences between groups.

Latency was analyzed with an additional mixed ANOVA with the same between groups measure as amplitude (3 levels: HC, HP, and NP) and one-within groups factor (noise—3 levels: SL, TN, and WN). *A priori* planned comparisons were conducted to determine group differences in latency during each task. For both amplitudes and latencies, effect sizes are stated using Hedges’*g* to account for uneven groups.

To assess the relationship between state hallucination ratings and noise condition, an ANOVA was performed with two within subject measures [1. noise condition (3 levels: SL, traffic, and WN) and 2. Hallucination state rating (3 levels: duration, loudness, and clarity)].

Correlational results were conducted to measure correlations between behavioral and demographic measures and MMN and P3a amplitudes. Bivariate Correlations (Spearmans’s rho) using a two-tailed significance level were run to analyse correlations between demographic and hallucination data with our ERP data. Additional correlations were performed between hallucination ratings and all scalp sites within each condition (SL, TN, and WN) to determine if there were any relationships between hallucinations experienced during the experiment in their duration, loudness and clarity to the P3a and MMN amplitude changes experienced.

## Results

### MMN Amplitude

There was a main effect of region, F(1,29) = 38.68 *p* < 0.001, due to larger amplitudes being shown in the frontal (M = −1.24 µV, SD = 1.12) compared to the central (M = −0.67 µV, SD = 0.97) regions. The effect of region was further shown when analyzing group differences by pairwise comparisons; HPs (M = −0.39 µV, SD= 1.06) showed a significant deficit over HCs (M = −0.89 µV, SD = 0.71; *p* = 0.029, *g* = 0.54), however, only for the central region. Finally, the frontal region showed significant differences between amplitudes during the SL (M = −1.34 µV, SD = 1.34) and TN (M = −0.88 µV, SD = 0.92, *p* = 0.029, *g* = 0.40) conditions, as well as the TN (M = −0.88 µV, SD = 0.92) and WN conditions (M = −1.51 µV, SD = 1.10, *p* = 0.001, *g* = 0.62).

**Figure 1:**
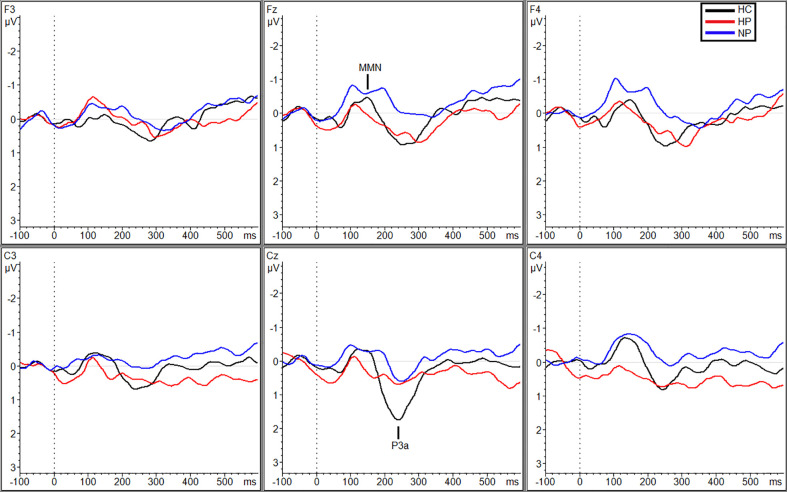
Grand averaged subtracted waveforms showing MMN and P3a across six scalp sites (F_3_, F_z_, F_4_, C_3_, C_z_, C_4_) for all participant groups (HC, HP, NP) during the silence condition.

While no main effect of group was shown, planned pairwise comparisons revealed significant differences at site C_4_ within the silent condition between HCs (M = −1.04, SD = 0.62) and HPs (M = −0.025, SD = 1.01, *p =* 0.013, *g* = 1.17) and HPs (M = −0.025 µV, SD =1.01) and NPs (M = −1.0 µV, SD = 0.86, *p* = 0.011, *g =* 1.04; **Figure 1**), no significant differences were observed between NPs and HCs.

**Figure 2:**
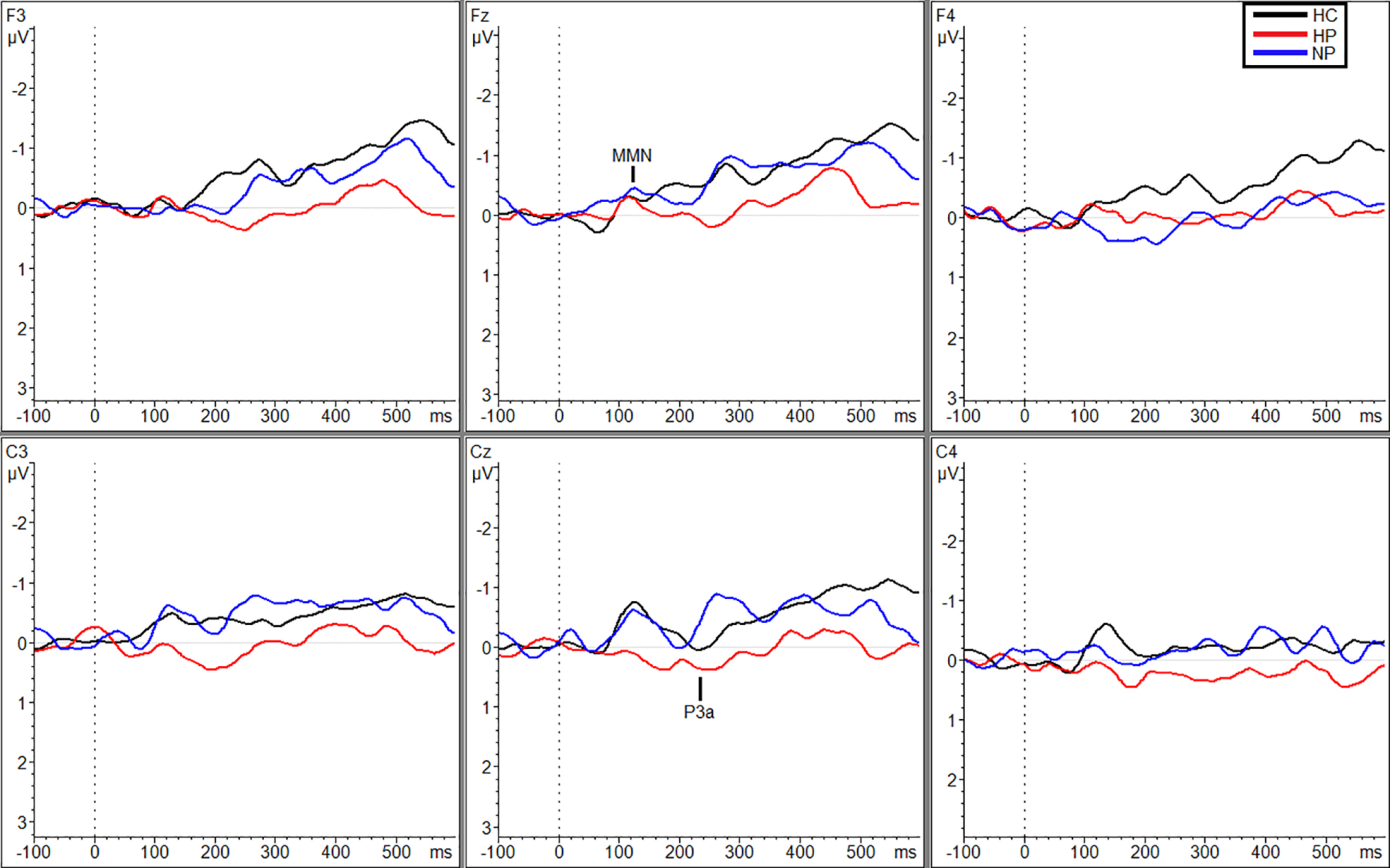
Grand averaged subtracted waveforms showing MMN and P3a across six scalp sites (F_3,_ F_z,_ F_4,_ C_3,_ C_z,_ C_4_) for all participant groups (HC, HP, NP) during the traffic condition.

**Figure 3:**
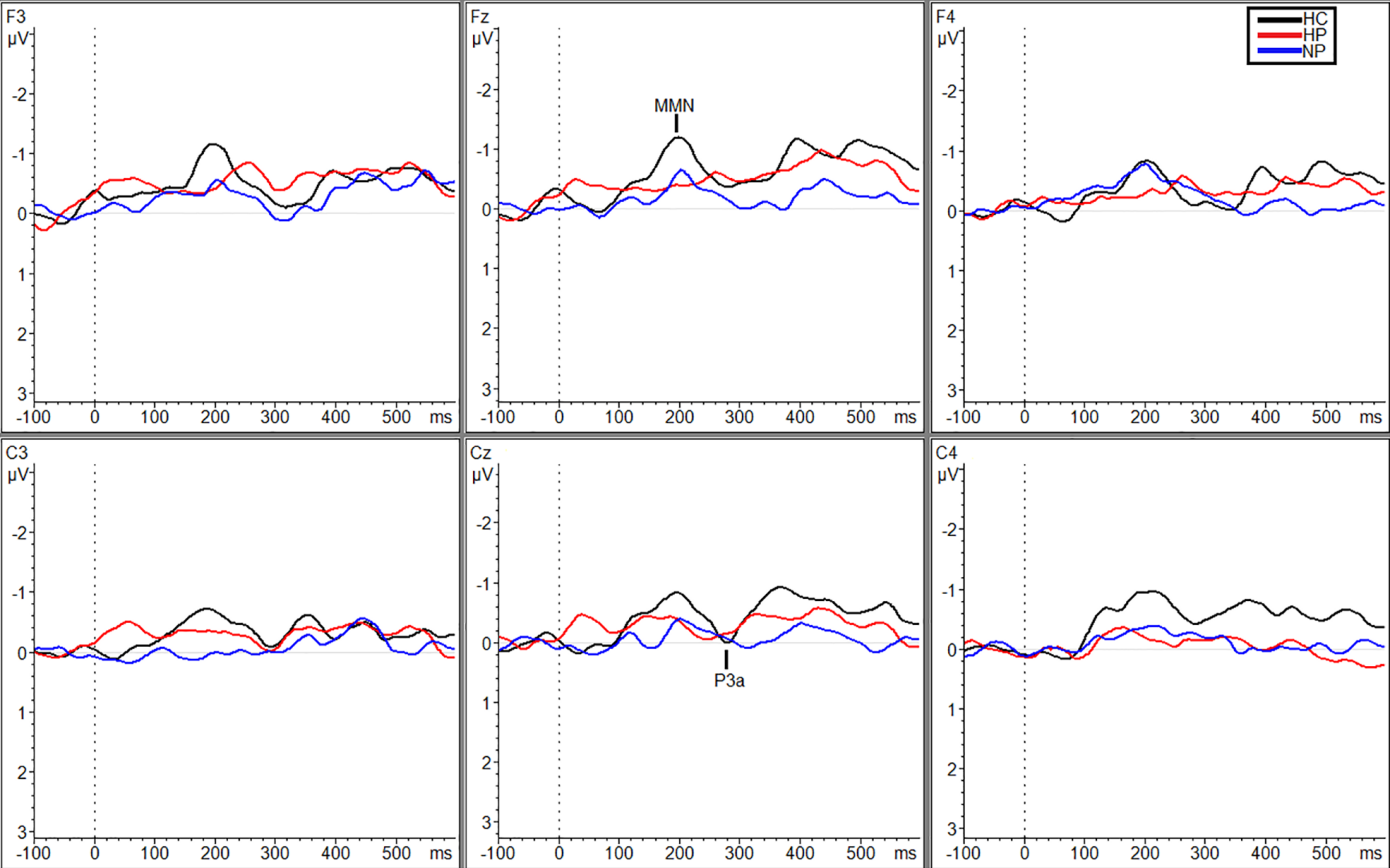
Grand averaged subtracted waveforms showing MMN and P3a across six scalp sites (F_3_, F_z_, F_4_, C_3_, C_z_, C_4_) for all participant groups (HC, HP, NP) during the white noise condition.

Additional significant differences resulting from pairwise comparisons were seen between HPs (M_Traf_ = 0.088 µV, SD_Traf_ = 0.80) and NPs (M_Traf_ = −0.98 µV, SD_Traf_ = 1.02; *p* = 0.010, *g* = 1.17) and HPs (M_Traf_ = 0.088 µV, SD_Traf_ = 0.80) and HCs (M_Traf_ = −0.79 µV, SD_Traf_ = 0.69; *p* = 0.039, *g* = 1.16) during the traffic condition however, only at site C_3_ (**Figure 2**). Additionally, at site C_z_, HPs (M_Traf_ = −0.31 µV, SD_Traf_ = 1.02) showed significant reductions in MMN amplitude compared to NPs (M_Traf_ = −1.11, SD_Traf_ = 0.74;*p* = 0.031; *g* = 0.89) in the traffic condition (**Figure 2**). Finally, significant differences were also observed at site C_4_ between HCs (M_WN_ = −1.28, SD_WN_ = 0.58) and the two patient groups (M_NP_ = −0.35, SD_NP_ = 0.66; *p* = 0.043, *g* = 1.49; M_HP_ = −0.27, SD_HP_ = 1.38; *p* = 0.027, *g* = 0.91) in the WN condition (**Figure 3**). See **Table 3** in supplemental materials for average MMN amplitudes within each group (HC, HP, and NP) under each noise condition (SL, traffic and WN).

### MMN Latencies

A main effect of condition was found, F(2, 58) = 10.93, *p* < 0.001, due to longer latencies in the WN (M = 199.19 ms SD = 41.03) compared to the SL (M = 156.70 ms, SD = 44.25) and traffic conditions (M = 162.18 ms, SD = 45.17).

### P3a Amplitudes

While there was no main effect of group, there were significant differences in the amplitudes present in HC between the SL condition (M = 1.39 µV, SD = 0.91) and the two noise conditions (M_Traf_ = 0.18 µV, SD_Traf_ = 0.82, *p* = 0.008, *g* = 1.40; M_WN_ = 0.25 µV, SD_WN_ = 0.93; *p* = 0.003, *g* = 1.24).

Planned comparisons showed larger amplitudes for HCs (M = 1.39 µV, SD = 0.91) compared to NPs (M = 0.34 µV, SD = 1.33; *p* = 0.022, *g* = 0.90), under the SL condition (**Figure 1**) and HPs (M = 0.77 µV, SD = 1.25) compared to NPs (M = 0.039 µV, SD = 0.82) during the TN condition (*p* = 0.036, *g* = 0.69; **Figure 2**). Followed up to individual sites, during the SL condition HCs exhibited larger amplitudes then the NPs at F_z_ (M_HC_ = 1.30 µV, SD_HC_ = 0.61; M_NP_ = −0.056 µV, SD_NP_ = 1.54; *p* = 0.028, *g* = 1.11) and C_z_ (M_HC_ = 2.70 µV, SD_HC_ = 1.63; M_NP_ = 1.22, SD_NP_ = 1.49; *p* = 0.032, *g* = 0.95; **Figure 1**). Additionally, HPs (M = 1.14 µV, SD = 1.42) exhibited a larger P3a than NPs (M = −0.056 µV, SD = 1.54) at site F_z_ during the SL condition, *p* = 0.035, *g* = 0.81. Finally, during the TN condition, HPs exhibited larger amplitudes then the NPs at F_3_ (M_HP_ = 0.97 µV, SD_HP_ = 1.03; M_NP_ = −0.24, SD_NP_ = 0.78; *p* = 0.003, *g* = 1.32) and F_z_ (M_HP_ = 0.97 µV, SD_HP_ = 0.91; M_NP_ = 0.19, SD_NP_ = 0.79; *p* = 0.019, *g* = 0.84; **Figure 2**). See supplemental materials for **Table 4** reporting P3a amplitude grand averages for each participant group (HC, HP, and NP) under each noise condition (SL, TN, and WN).

### P3a Latencies

There was a main effect of task found F(2,58) = 4.94, *p* = 0.010 due to longer latencies being present during the WN condition (M = 286.57 ms, SD = 51.83) compared to the other two conditions (M_silence_ = 256.79 ms, SD_silence_ = 47.39; M_Traf_ = 258.37 ms, SD_Traf_ = 54.29) conditions. *Follow up* comparisons revealed this was limited to the HC condition, *p*-values ranging from *p* = 0.021 to *p* = 0.007.

### MMN Correlations

PANSS negative symptom scores were positively correlated (i.e., decreased MMN amplitude with increased PANSS score) at site F_4_ during the traffic condition (r = .42, p = .043).

PSYRATS scores were positively correlated (i.e., decreased MMN amplitude with increased PSYRATS score) at sites C_3_ (r = .46, p = .028) and C_Z_ (r = .42, p = .048) in the traffic condition.

Age was also positively correlated with frontal site F_4_ in the WN condition (*r* = 0.49, *p* = 0.004). Finally, the neuroleptic dosage (as measured in chlorpromazine equivalents) was significantly correlated with F_4_ (*r* = 0.42, *p* = 0.048) in the SL condition. Additionally, a negative correlation (r = −.49, p = .018) was present between the latency during the WN condition and neuroleptic dosage.

Additional bivariate correlational analysis using spearman’s rho was used to examine the relationship between duration, loudness and clarity of any hallucinations present during the experiment and their correlation with MMN amplitude at all sites separated by condition. There were no significant findings between measures of hallucinatory state and MMN amplitude.

### P3a Correlations

Age was significantly correlated with C_3_ (*r* = −0.36, *p* = 0.043) in the SL condition. Neuroleptic dosage was found to significantly correlate with P3a amplitude at C_3_ (*r* = 0.52, *p* = 0.010), however, only in the SL condition.

**Figure 4:**
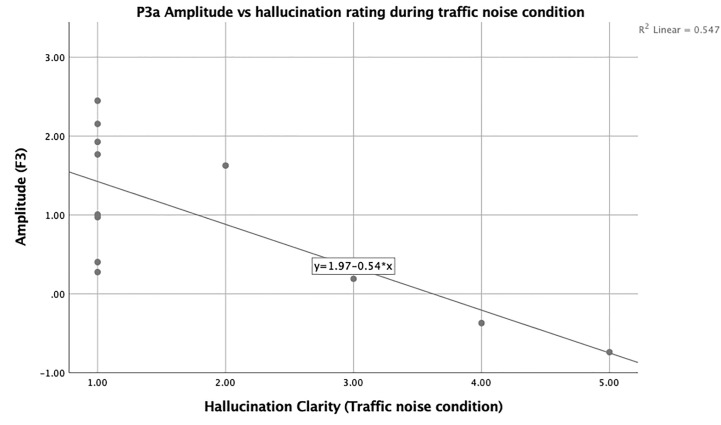
Scatter plot showing the negative relationship between F_3_ and state hallucination clarity in hallucinating participants.

Additional bivariate correlational analysis using Spearman’s rho was used to examine the relationship between duration, loudness, and clarity of any hallucinations present during the experiment and their correlation with P3a amplitude at all sites separated by condition. There were significant correlations between the clarity (*r* = −0.69, *p* = 0.013; **Figure 4**) of hallucinations and amplitude at site F_3_ during the TN condition.

### Hallucination State Ratings

There was no main effect of noise condition on hallucination state ratings F(2,22) = 0.55, *p* = 0.58.

## Discussion

This study assessed the relationship between MMN and P3a amplitudes and latencies in a SZ sample with and without treatment-resistant AHs. Not surprising, MMN amplitude was decreased overall in both patient samples when compared to HCs which has been a consistent finding across the literature (24, 36, 42–44, 54, 95, 96). Interestingly, this finding was only significant for the HP group in comparison to the NP group and, furthermore, degree of MMN reduction was associated with trait-level markers of AH severity (as indexed by the PSYRATS). These findings are consistent with work by Ford et al. (84), which showed that Brodmann area 41 (i.e., transverse temporal gyri of Heschl), the temporal site of MMN generation, was functioning at a higher level in non-hallucinators compared to the hallucinators.

Furthermore, decreases in amplitude tended to be condition specific; more specifically, while HPs had significantly reduced amplitudes in comparison to NPs and HCs, this finding was restricted to the traffic and WN conditions. This suggests that the brain of individuals with AHs may have an overall harder time processing changes in phonetic stimuli when background noise is present, perhaps due to structural or functional differences in cortical function that underlie AHs. Furthermore, when background noise becomes more complex or irregular (TN condition) individuals with AH may struggle with secondary cortical processing as opposed to during the SL conditions (97). It has been previously shown that AHs are reduced when external speech is present (20–22), with a corresponding deactivation of auditory cortex suggesting generalized cortical hypofunction due to sensory overload when AHs compete with external auditory stimulation (98, 99). Our findings suggest that auditory processing network is more taxed in the HP group during the traffic condition in comparison to all other conditions, and based on our correlations, it does not appear that there is a lack of resources present in this group but rather that this could outline a deficit in automatic primary or secondary cortical processing in a SZ sample. This is only the case, however, for those experiencing AH, which represent a specific neurological challenge faced by this particular sample. In addition to AHs, MMN amplitude was reduced in those clinical participants with greater PANSS negative symptom scores, consistent with previous work in both those with SZ (100) and a ketamine model of SZ in healthy participants (101). In future studies, it would be beneficial to include a time course component to measure the onset and offset of AHs for the duration of each task, as this would allow for further analysis on how state AHs impact MMN and P3a amplitude and latency.

Previous studies have analyzed background noise and its overall effects on cognitive processing in individuals with SZ; one study in particular used urban and social noise conditions to determine the cognitive burden on patients (102). Both the social and urban noise conditions were comparable to our TN condition. Wright et al. (102) found that patients had significantly reduced psychomotor speed, attention, working, and verbal memory in the presence of background noise from both of their conditions. The presence of background noise inhibiting the ability of verbal and working memory could play a part in the brain’s ability to detect differences, whether that be through comparison to a memory trace or a predictive model of the auditory environment. Furthermore, verbal memory has been shown to be impacted in MMN processing when phonemic stimuli are used (25, 54, 103, 104). Additionally, Dittmann-Balcar, Thienel, and Schall (105) found that MMN was reduced in participants if they were simultaneously performing an auditory discrimination task. While our participants were told to ignore the auditory tones being presented, this could support our finding of seeing significant reductions in the TN condition for our HPs—due to the nature of the TN and WN conditions being more auditorily demanding than the SL condition. It could also have to do with the fact that individuals with SZ are known to have complications with weighting and processing incoming auditory stimuli, likely due to a delay in the brain’s ability to transmit and process incoming stimuli and then act on said stimuli (23).

P3a amplitudes were significantly larger in the SL background noise condition when compared to both active noise conditions (TN and WN); however, this was most prominent in the NPs. In contrast to HPs, who showed specific deficits in early auditory change processing during the presence of background noise, this suggests that NPs are impaired in later attention switching or stimuli categorization processes when background noise is applied. This finding appears to conflict with the findings of Fisher et al. (77) which showed the largest P3a deficits in the HP condition; however, this study only examined P3a response to single-vowel phonemes under conditions of no background noise.

While there was no association between P3a amplitudes and hallucinatory trait (either *via* group status or correlations with the PSYRATS), clarity of state hallucinations was negatively correlated with P3a amplitude at frontal site F_3_ during the traffic condition while MMN amplitude was not correlated with state hallucination ratings. This suggests that the P3a is more sensitive to hallucinatory state, while the MMN appears to be more strongly linked to hallucinatory trait within our sample, similar to previous reports by our group in an acutely ill sample (64).

## Limitations and Summary

This study does not go without its limitations, most notably the small sample size and the modest level of hallucinatory activity expressed by our SZ patients; it is unclear whether more severe hallucinatory activity would produce a stronger activation with background noise. While it is difficult to induce hallucinations, future studies may wish to recruit participants with more severe histories of AHs. In addition to this, the presence of active hallucinations varied in our HP participants during the three noise conditions; in future studies, it would be beneficial to measure those actively experiencing AHs vs. those who are not experiencing active hallucinations. This would allow for a more clear understanding on the impact of active AHs on MMN and P3a amplitude and latency. Furthermore, the medicated status of the patients could have limited the observations, although there was no significant difference in neuroleptic dosage between SZ groups. Future studies may also wish to incorporate structural neuroimaging techniques, such as magnetic resonance imaging and/or diffusion tensor imaging, in order to explore the potential underlying neuroanatomical correlates of observed deficits in acoustic change detection. A final limitation is the retrospective measure of hallucinatory state; a better method may be to ask participants to signal the onset and offset of hallucinations during stimulation.

Nevertheless, the study did find interesting and novel findings suggesting those with SZ and treatment-resistent AHs show earlier (i.e., MMN) deficits in the context of background noise, while these same conditions affect later processes (i.e., P3a) more strongly in those without AHs. The P3a findings contradicted our hypotheses, as it was anticipated that the HPs would show the greatest deficit on all measures. Overall, this suggests that the presence or absence of persistent AHs is associated with differing function of the auditory processing stream, with important implications for personalized treatments.

## Data Availability Statement

The datasets generated for this study will not be made publicly available because participants did not consent to the sharing or public availability of data.

## Ethics Statement

The studies involving human participants were reviewed and approved by Research Ethics Board of the Royal Ottawa Mental Health Centre and Department of Psychology Research Ethics Board, Carleton University. The patients/participants provided their written informed consent to participate in this study.

## Author Contributions

AF wrote all drafts of the manuscript and analyzed the data set. DF contributed to study design, collected all data, and contributed to analysis and interpretation of finding, as well as editing all drafts. VK and AL contributed to design of study and editing of final draft of the manuscript.

## Conflict of Interest

The authors declare that the research was conducted in the absence of any commercial or financial relationships that could be construed as a potential conflict of interest.
