# Supplementary material for: Interaction of Background Noise and Auditory Hallucinations on Phonemic Mismatch Negativity (MMN) and P3a Processing in Schizophrenia
Source: Front Psychiatry. 2020 Sep 15;11:540738. doi: 10.3389/fpsyt.2020.540738 (PMC7523538; doi:10.3389/fpsyt.2020.540738)
Supplement: Supplementary file 1 [file DataSheet_1.pdf]

Supplemental findings:

3.1 MMN amplitudes

There was no main effect of background noise, however, there was a significant difference found between the traffic condition (M= -0.73 μV, SD= 0.91) and white noise conditions (M= -1.13 μV, SD= 1.00, *p*= 0.014, *g*=0.42). Follow up tests revealed the difference between the traffic (M= -0.47 μV, SD= 1.07) and white noise conditions (M=-1.09 μV, SD=1.11) were limited to the HP participants, (*p*=0.017, *g*=0.57), specifically at two sites F_3 (M_{Traf} =- 0.52 μV, SD_{Traf} = 1.23; and M_{WN} = -1.60 μV, SD_{WN} = 0.99, *p*=0.001, *g*=0.97) and F_z (M_{Traf} = -0.86 μV, SD_{Traf} =1.26, M_{WN} = -1.66 μV, SD_{WN} = 1.13, *p*=0.024, *g*=0.67).

3.2 MMN Latencies

Follow up comparisons were completed to further investigate whether this difference was observed in all groups; comparisons showed significant differences within HCs in the silence (M= 150.00 ms, SD= 35.90) and white noise (M= 205.56 ms, SD= 30.05, *p*=0.004, *g*=1.68) conditions; further differences were shown between the traffic (M= 162.22 ms, SD= 41.89) and white noise conditions (M= 205.56 ms, SD=30.05, *p*=0.020, *g*= 1.19). Finally, there was a significant difference in the latency of NPs during the silence (M= 157.27 ms, SD= 52.95) and white noise condition (M= 215.80 ms, SD= 35.28, *p*=0.001,*g*= 1.30), and during the traffic (M= 173.80, SD= 54.48) and white noise conditions (M= 215.80 ms, SD= 35.28, *p*=0.014, *g*= 0.92).

3.3 P3a amplitudes

There was a main effect of condition, F(2,58) = 6.54, *p*= 0.003, with larger amplitudes in the silence (M= 0.89 μV, SD= 1.30) compared to the traffic (M= 0.33 μV, SD= 1.04) and white noise (M= 0.24 μV, SD= 1.07) conditions. Followed up to account for region, the findings

between the silence (M= 1.12, SD= 1.31) and traffic noise (M= 0.39, SD=0.97) conditions were limited to the central region (*p*=0.008, *g*=0.63). While the differences between the silence and white noise conditions were found at both frontal ($M_{silence}$=0.67 $SD_{silence}$=1.28; M_{WN}=0.075 SD_{WN}=1.22; *p*=0.019, *g*=0.49) and central sites ($M_{silence}$=1.12, $SD_{silence}$= 1.31; M_{WN}=0.40, SD_{WN}= 0.93; *p*=0.006, *g*= 0.63).

HCs showed significant differences between the silence condition and the two noise conditions at F_z and all central sites with *p*- values ranging from *p*<0.046 – *p*<0.001. Additional amplitude differences were shown in HPs between the silence (M= 0.96 μV, SD= 1.41) and white noise condition (M= 0.086 μV, SD= 1.08; *p*=0.007, *g*=0.70), as well as during between the traffic (M= 0.76, SD= 1.29) and white noise conditions (M= 0.086, SD= 1.08; *p*=0.025, *g*= 0.57), however, only at the frontal sites *p*-values ranging from *p*<0.018 – *p*<0.001.

There was also a main effect of region found, F(1, 29)= 7.34, *p*= 0.011, due to a larger amplitude at central regions (M= 0.64 μV, SD= 1.07) compared to frontal (M= 0.34 μV, SD=1.20) regions.

Supplemental Tables:

Table 3:
a) Grand average waveforms showing MMN amplitude elicited during the silence condition at frontal and central sites for healthy controls (HC), hallucinating participants (HP) and non-hallucinating participants (NP)
b) Grand average waveforms showing MMN amplitude elicited during the traffic condition at frontal and central sites for healthy controls (HC), hallucinating participants (HP) and non-hallucinating participants (NP)
c) Grand average waveforms showing MMN amplitude elicited during the white noise condition at frontal and central sites for healthy controls (HC), hallucinating participants (HP) and non-hallucinating participants (NP)

A)

	Silence Condition					
	F3	Fz	F4	C3	Cz	C4
HC	-1.37	-1.84	-1.68	-0.52	-0.78	-0.98
HP	-1.91	-1.23	-1.68	-0.60	-0.24	-0.23
NP	-1.37	-1.95	-1.62	-0.77	-1.18	-0.93

B)

	Traffic Condition					
	F3	Fz	F4	C3	Cz	C4
HC	-1.18	-1.40	-1.27	-0.66	-0.95	-0.31
HP	-1.20	-1.33	-1.64	-0.89	-1.25	-0.70
NP	-0.83	-1.29	-0.82	-0.014	-0.48	-0.46

C)

	White Noise Condition					
	F3	Fz	F4	C3	Cz	C4
HC	-1.89	-1.98	-1.69	-0.78	-1.30	-1.27
HP	-1.68	-1.68	-1.52	-0.48	-0.59	-0.41
NP	-2.02	-1.74	-1.30	-0.91	-0.93	0.048

Table 4:
a) Grand average waveforms showing P3a amplitude elicited during the silence condition at frontal and central sites for healthy controls (HC), hallucinating participants (HP) and non-hallucinating participants (NP)
b) Grand average waveforms showing P3a amplitude elicited during the traffic condition at frontal and central sites for healthy controls (HC), hallucinating participants (HP) and non-hallucinating participants (NP)
c) Grand average waveforms showing P3a amplitude elicited during the white noise condition at frontal and central sites for healthy controls (HC), hallucinating participants (HP) and non-hallucinating participants (NP)

A)

	Silence Condition					
	F3	Fz	F4	C3	Cz	C4
HC	0.67	1.30	1.16	1.26	2.70	1.25
HP	0.57	1.14	0.90	0.92	1.087	1.13
NP	0.19	-0.055	0.16	0.067	1.22	0.066

B)

	Traffic Condition					
	F3	Fz	F4	C3	Cz	C4
HC	-0.058	0.23	0.11	-0.073	0.66	0.22
HP	0.97	0.97	0.51	0.45	0.98	0.70
NP	--0.23	-0.19	0.12	-0.075	0.48	0.13

C)

	White Noise Condition					
	F3	Fz	F4	C3	Cz	C4
HC	-0.026	0.34	0.52	-0.041	0.65	0.062
HP	-0.44	-0.12	-0.28	0.13	0.76	0.45
NP	0.26	0.52	-0.11	0.35	1.10	0.13

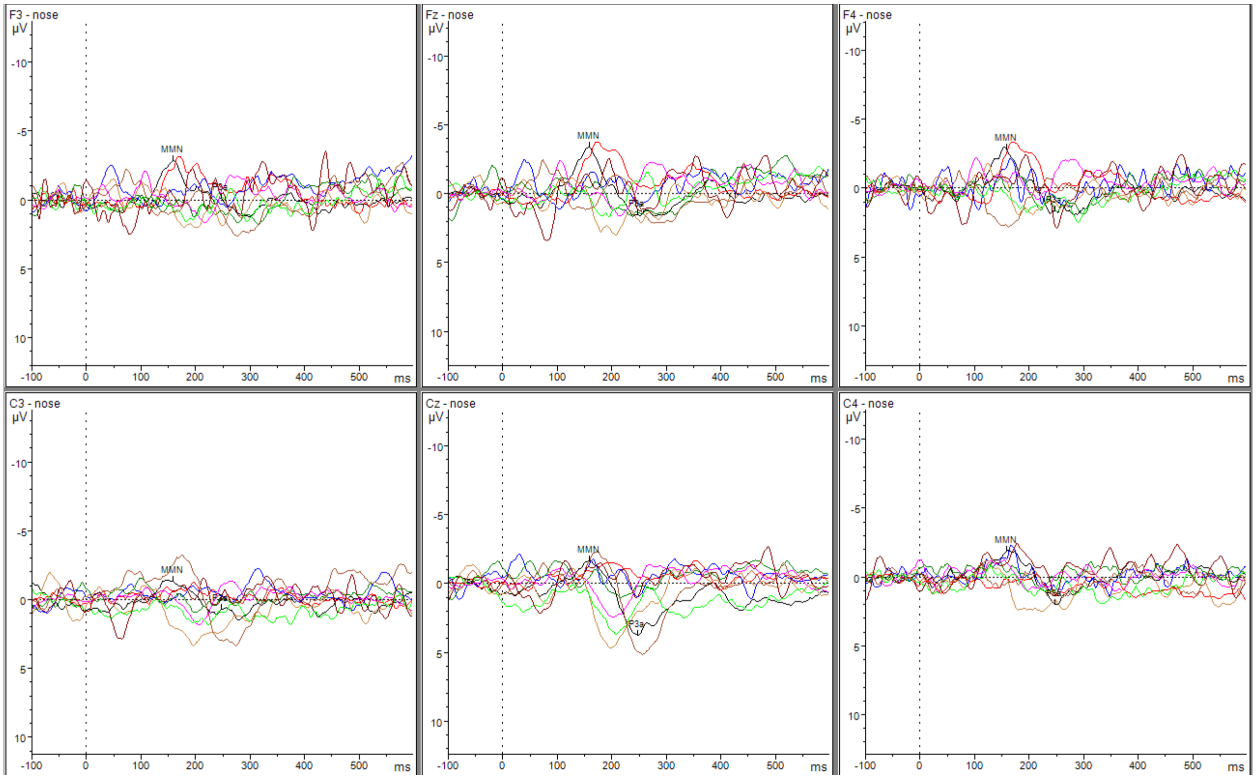

Figure 5. Graph depicting the average MMN and P3a for each participant in the HC group for the silence condition shown across six scalp site ($F_{3,}$ $F_{z,}$ $F_{4,}$ $C_{3,}$ $C_{z,}$ C_{4}).

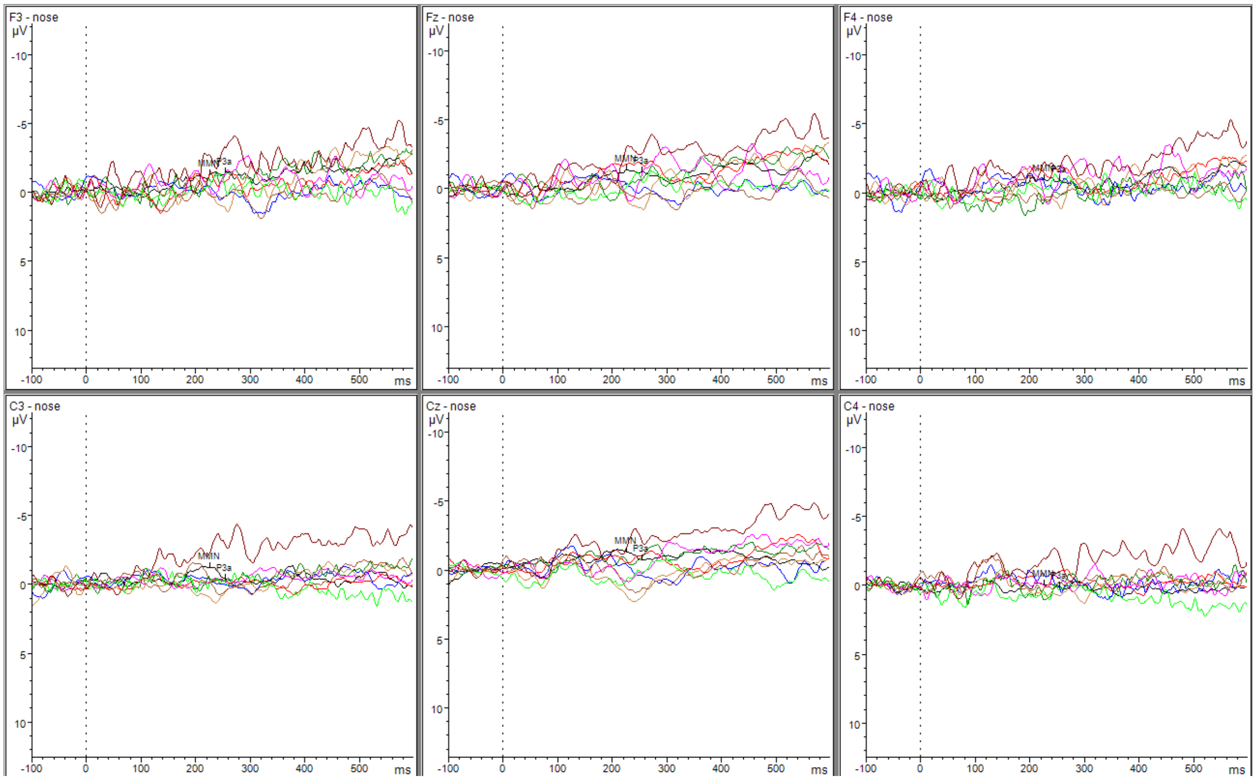

Figure 6. Graph depicting the average MMN and P3a for each participant in the HC group for the traffic condition shown across six scalp site ($F_{3,}$ $F_{z,}$ $F_{4,}$ $C_{3,}$ $C_{z,}$ C_4).

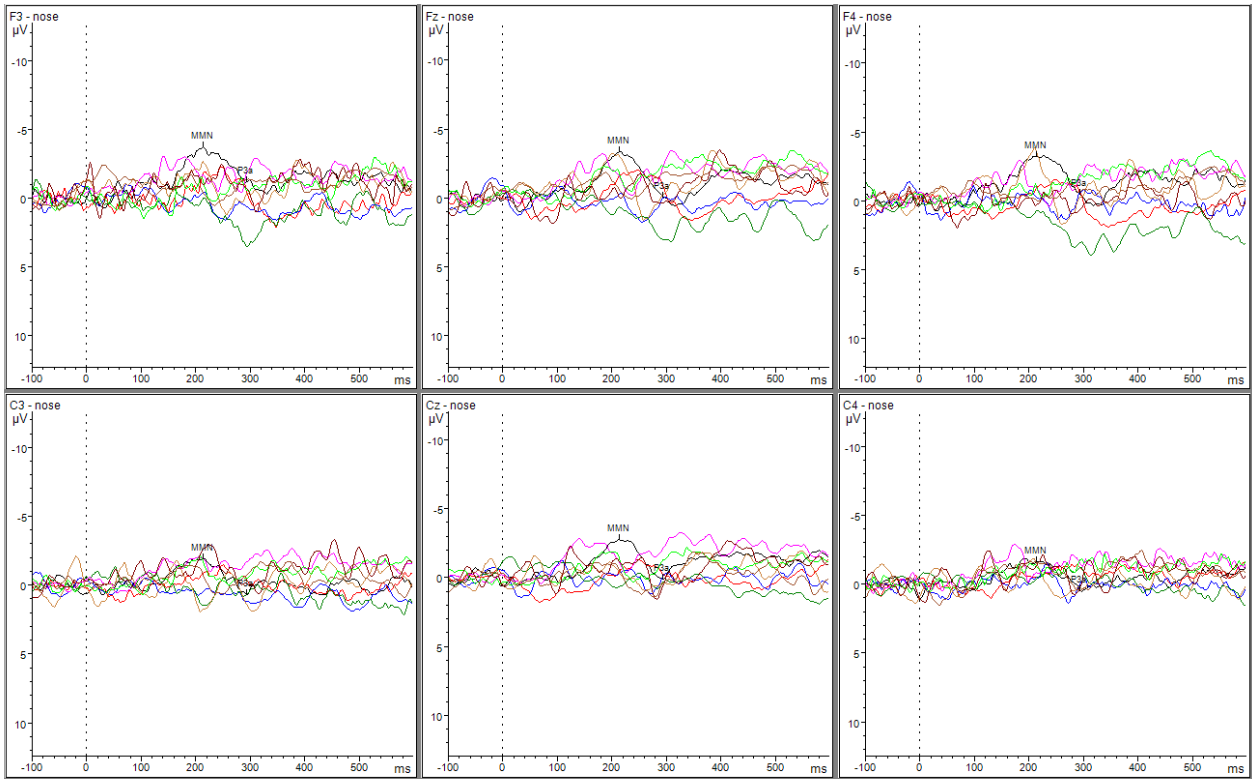

Figure 7. Graph depicting the average MMN and P3a for each participant in the HC group for the white noise condition shown across six scalp site (F_3, F_z, F_4, C_3, C_z, C_4).

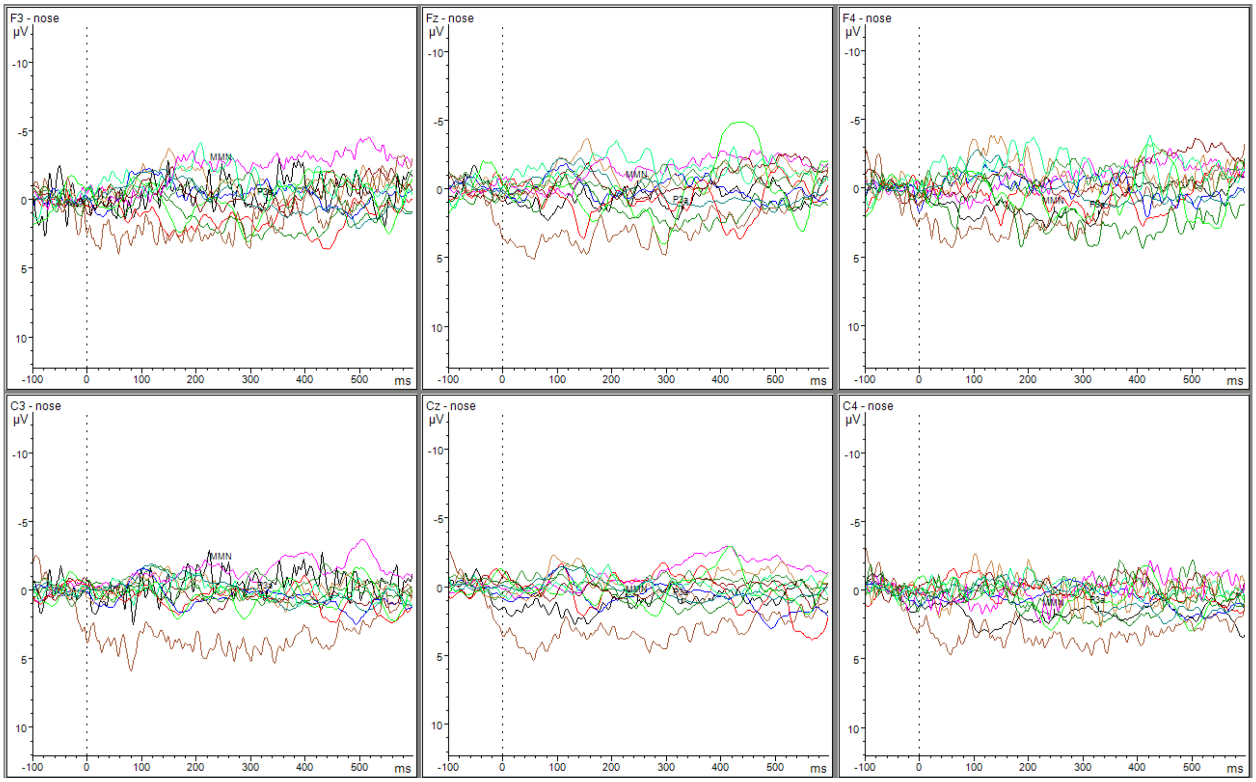

Figure 8. Graph depicting the average MMN and P3a for each participant in the HP group for the silence condition shown across six scalp site (F_3, F_z, F_4, C_3, C_z, C_4).

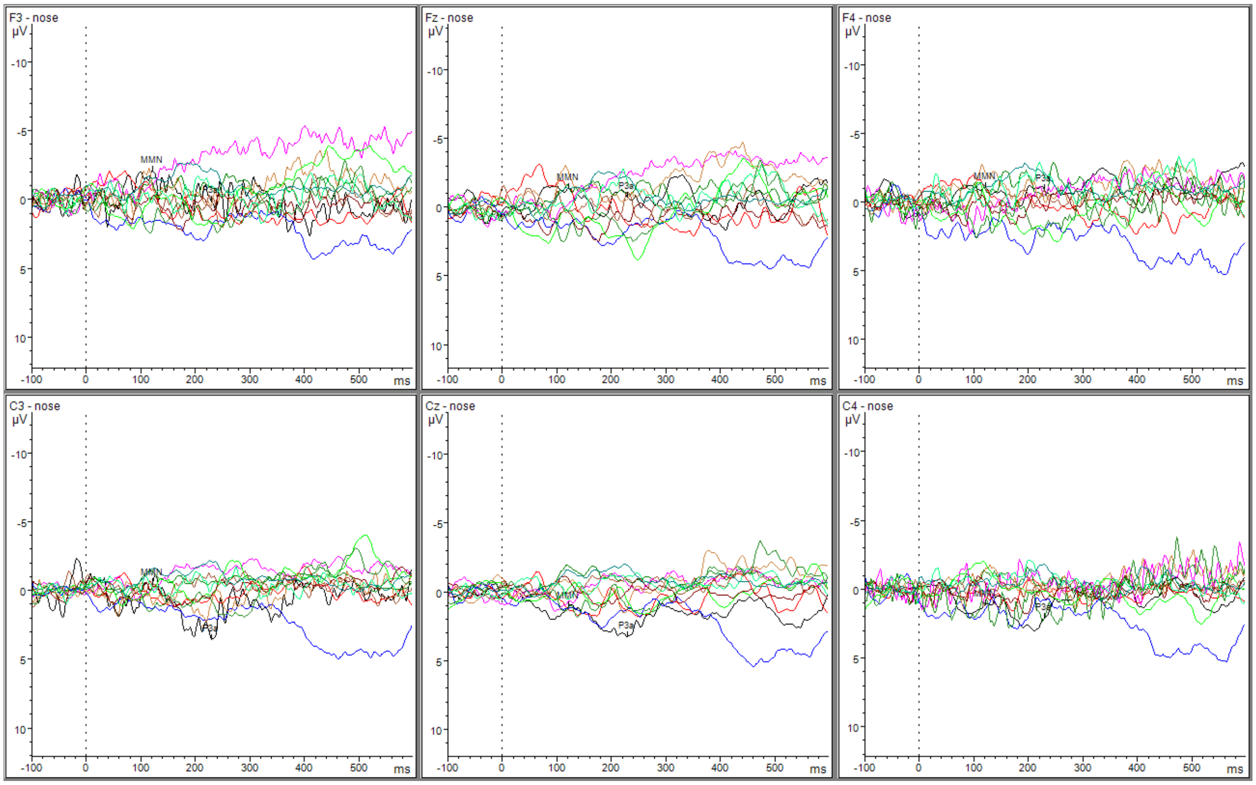

Figure 9. Graph depicting the average MMN and P3a for each participant in the HP group for the traffic noise condition shown across six scalp site (F_3, F_z, F_4, C_3, C_z, C_4).

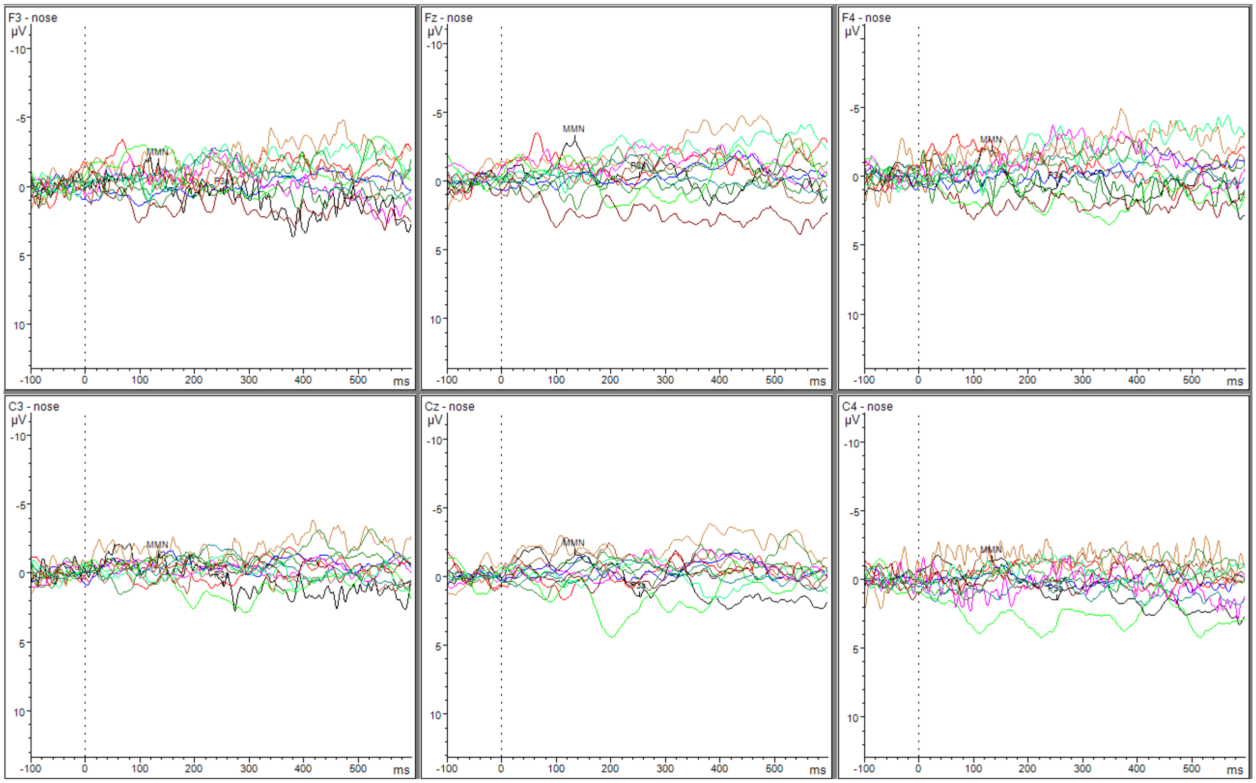

Figure 10. Graph depicting the average MMN and P3a for each participant in the HP group for the white noise condition shown across six scalp site ($F_{3,}$ $F_{z,}$ $F_{4,}$ $C_{3,}$ $C_{z,}$ C_4).

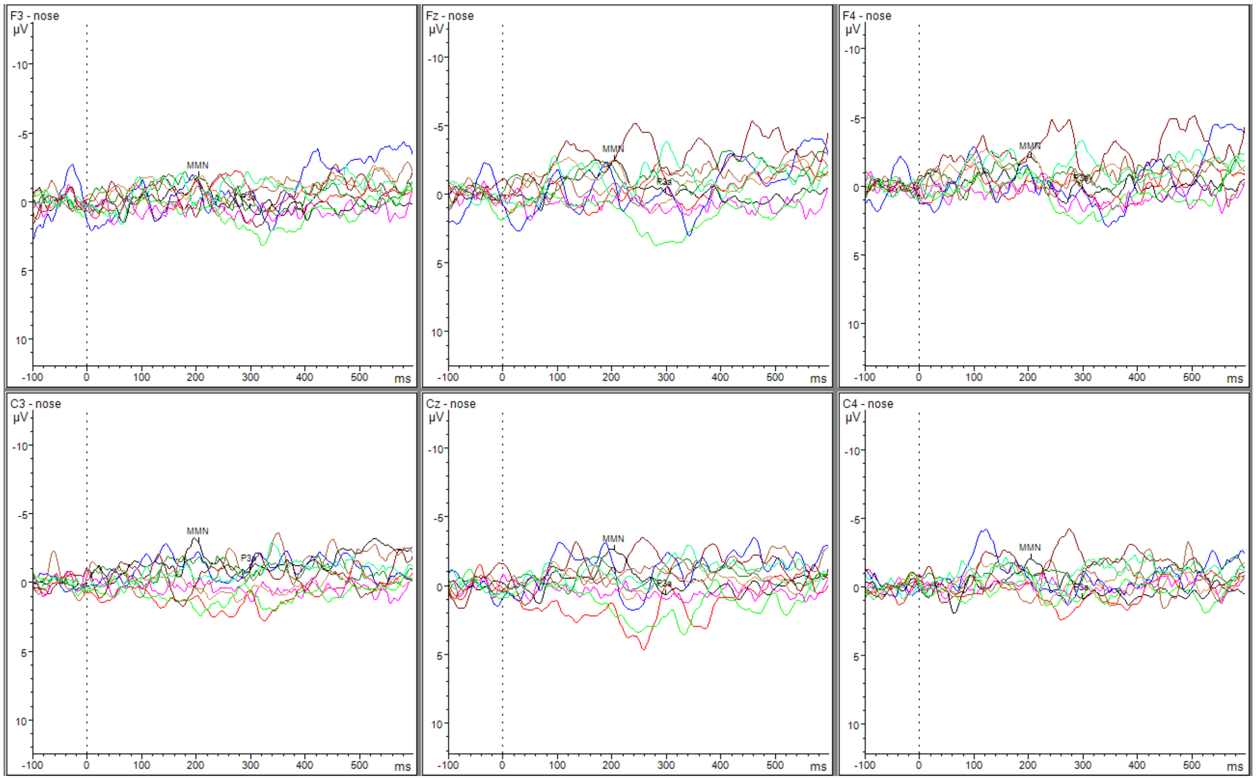

Figure 11. Graph depicting the average MMN and P3a for each participant in the NP group for the silence condition shown across six scalp site ($F_{3,}$ $F_{z,}$ $F_{4,}$ $C_{3,}$ $C_{z,}$ C_4).

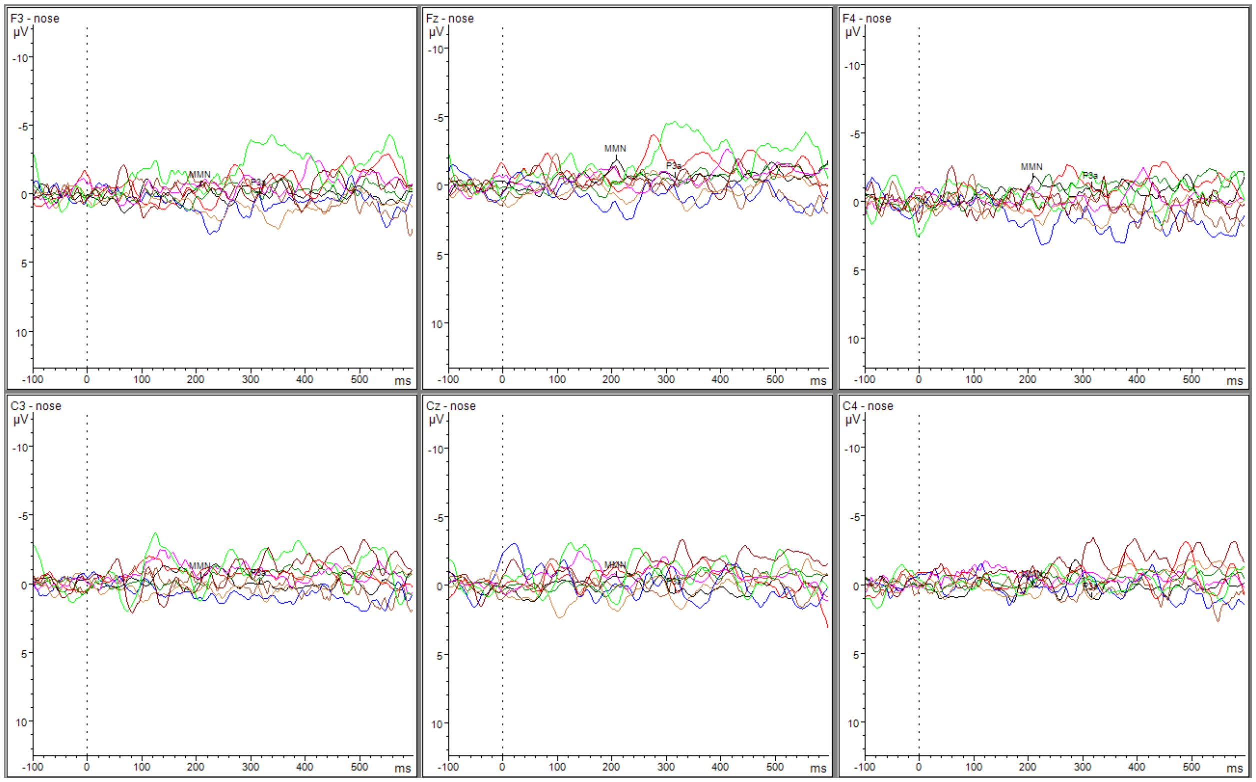

Figure 12. Graph depicting the average MMN and P3a for each participant in the NP group for the traffic noise condition shown across six scalp site (F_3, F_z, F_4, C_3, C_z, C_4).

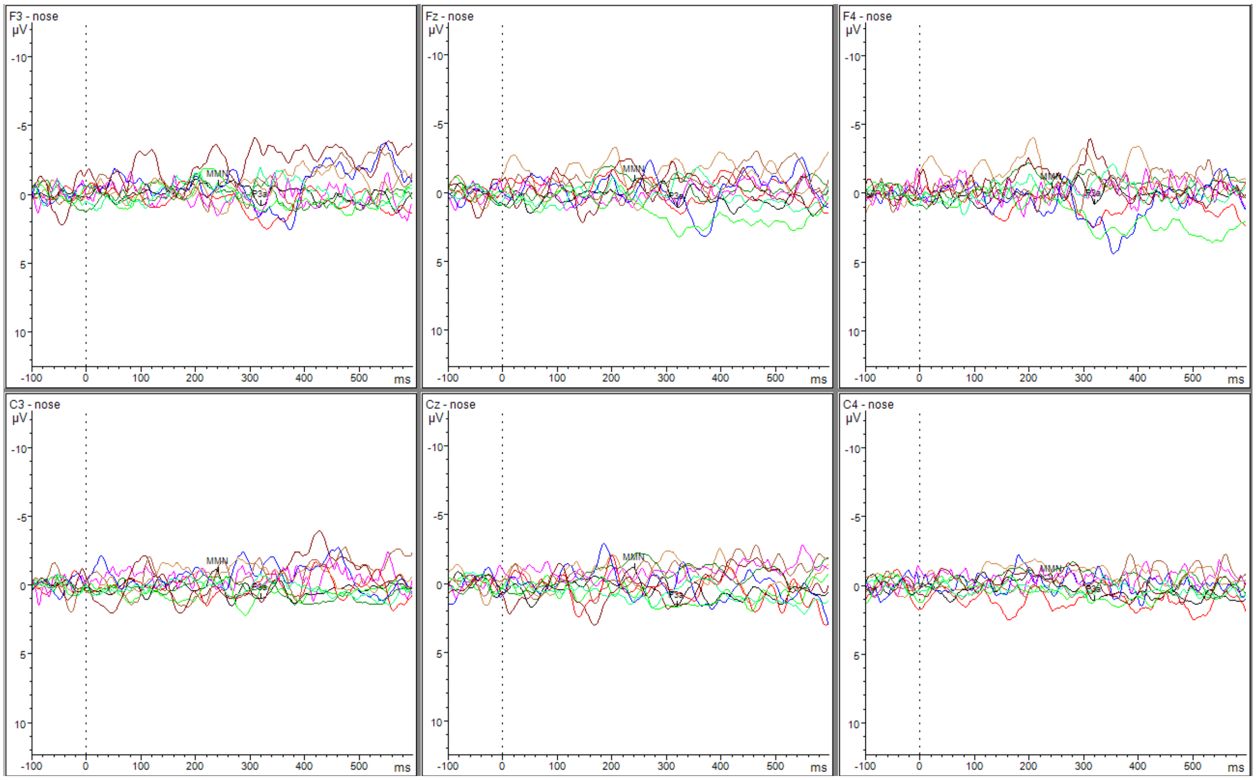

Figure 13. Graph depicting the average MMN and P3a for each participant in the NP group for the white noise condition shown across six scalp site (F_3, F_z, F_4, C_3, C_z, C_4).
